# An update in clinical utilization of photodynamic therapy for lung cancer

**DOI:** 10.7150/jca.51537

**Published:** 2021-01-01

**Authors:** Kai Wang, Boxin Yu, Janak L. Pathak

**Affiliations:** 1 International Medicine Center, Tianjin Hospital, 406 south of JieFang road, HeXi District, Tianjin, China; 2 Affiliated Stomatology Hospital of Guangzhou Medical University, Guangzhou Key Laboratory of Basic and Applied Research of Oral Regenerative Medicine, Guangzhou 510182, China

**Keywords:** Photodynamic therapy, lung cancer, inoperable cancer, cancer treatment

## Abstract

Lung cancer is one of the leading causes of cancer-related death worldwide, with nearly 1.8 million-diagnosis and 1.59 million deaths. Surgery, radiotherapy, and chemotherapy in individual or combination are commonly used to treat lung cancers. Photodynamic therapy (PDT) is a highly selective method for the destruction of cancer cells by exerting cytotoxic activity on malignant cells. PDT has been the subject of numerous clinical studies and has proven to be an effective strategy for cancer therapy. Clinical studies revealed that PDT could prolong survival in patients with inoperable cancers and significantly improve quality of life. For inoperable lung cancer cases, PDT could be an effective therapy. Despite the clinical success reported, PDT is still currently underutilized to treat lung cancer and other tumors. PTD is still a new treatment approach for lung cancer mainly due to the lack of enough clinical research evaluating its' effectiveness and side effects. In this review, we discuss the current prospects and future potentials of PDT in lung cancer treatment.

## Introduction

Lung cancer is the most prevalent cancer in the world, with nearly 2.1 million diagnoses and 1.8 million deaths in 2018 1. Surgery is the first choice of intervention for early-diagnosed lung cancers 2. However, surgery is difficult at the late stage-diagnosed lung cancer due to uncontrolled invasion and metastasis. More than 20% of early-diagnosed lung cancers are inoperable due to patient old age, severely impaired lung function, and other comorbidities 3. Stereotactic body radiotherapy, cryotherapy, and systemic chemotherapy have been introduced for lung cancer therapy 4. But these treatment approaches have a significant risk of systemic adverse effects. Photodynamic therapy (PDT) is an ablative therapy that kills cancer cells by photosensitizing the targeted tumor with visible light exposure with a specific wavelength 5. External-beam radiation therapy delivers ionizing radiation, but PDT delivers non-ionizing electromagnetic irradiation. PDT consists of 3 essential components, i.e., photosensitizer, light, and oxygen, that induce focal cell death without exerting systemic adverse effects. PDT has been used to treat various cancers, including lung, head, and neck, brain, pancreas, intraperitoneal cavity, breast, prostate, skin, and liver 6-8. PDT had shown the potential to treat minimally invasive lung cancer, especially the central type of early-stage lung cancer 9-16. In the case of inoperable disease and failure or refusal of other treatments, PDT can be a choice of therapy or part of the combination therapy for lung cancer. However, PDT is still currently underutilized to treat lung cancer and other tumors in clinics. In this review, we discuss the clinical application of PDT in different types of lung cancers, the existing problems, and the possible troubleshoot for effective lung cancer treatment.

## The history of PDT

The history of PDT has been well described in previous literature 6, 17, 18. Over 3000 years ago, ancient Egyptian, Chinese, and Indian civilizations already used light in combination with reactive chemicals to treat conditions like vitiligo, psoriasis, and skin cancer 17, 19. More than 30 years ago, PDT was clinically approved for treating a small number of selected tumors 20. Besides cancer, PDT has been used in cardiology 21, 22, urology 23, immunology 24, ophthalmology 25, 26, dentistry 27, dermatology 28, 29, and cosmetics 30, 31. Although the US Food and Drug Administration (FDA) approved PDT for clinical use 25 years ago, it remains underutilized clinically. This review addresses its clinical application status and outlook by summarizing the biological and physicochemical aspects.

## Basic concept and mechanisms of PDT

The basic concept and mechanism of PDT are well described in the previous review papers 32-34. PDT is a form of non-ionizing radiation therapy that uses a photosensitizer, combined with light to produce singlet oxygen. Singlet oxygen can exert anti-cancer activity through apoptotic, necrotic, or autophagic tumor cell death 35-37. Photosensitizer drug accumulates (actively or passively) in the specific tumor sites. Once photosensitizer is adsorbed in tumor tissue, it can be excited by appropriate wavelength laser irradiation 33, 38. During laser irradiation, photon absorption by ground state photosensitizer activates to excited singlet state. A change in the spin of electrons known as intersystem crossing converts the singlet state to a triplet state that interacts with surrounding molecules to produce reactive oxygen species (ROS) via two different processes 39, 40. In the first process, the transfer of a hydrogen atom to an electron between the excited photosensitizer and substrates leads to the generation of free radicles that react with oxygen, producing ROS such as superoxide and hydroxyl radicles 39. Photosensitizers have couples of electrons with opposite spins in low-energy molecular orbitals. Inversion of the electron spin is the reason for the relatively long life (microseconds) of the excited triplet state, which can be involved in two types of processes. In a type I process, the photosensitizer abstracts an electron from a reducing molecule in its vicinity. In type II photoreaction, photosensitizer transfers its energy directly to molecular oxygen. Besides, biological systems are enzymatically protected against superoxide 41. This two-stage procedure significantly reduces side effects, as the harmless photosensitizer is activated only via directed illumination, resulting in local tissue destruction**.** The choice of optimal combinations of photosensitizers, light sources, and treatment parameters are crucial for effective PDT 42, 43. The type of photosensitizer used, oxygen concentration within target tumor cells, a dose of light applied, and concentration of photosensitizer uptaken by cancer cells determine the cancer treatment efficacy of PDT 44-47. PDT components can directly induce cellular damage to organelles and cell membranes, depending on where they are generated 21, 22. High selectivity of the treatment site is the main advantage of PDT 39.

## Mechanism of PDT-induced cell death

The alone or simultaneous combined effect of apoptosis, autophagy, mitoptosis, or necrosis alone events is the main pathway leading to PTD-induced cancer cell death 48-52. Liu X and colleagues reported that the release of cytochrome c from mitochondria as a route of the PDT-induced cancer cell apoptosis 53. Mitochondria are the common target for PDT and photosensitizer inducing mitochondrial disintegration causes apoptosis 54. The direct effect of PDT on caspases, BCL2 protein family members, and apoptosis-inducing factors triggers apoptosis 34. Photosensitizer localization in the plasma membrane and nuclei of target lung cancer cells induces a necrotic form of cell death 55. PDT-mediated cancer control is also associated with the effect of photodamage to the tumor vasculature and the enhanced anticancer immunogenic responses 56-59.

## PDT in oncology

The mechanism of PDT-mediated antitumor activity has been examined for the past several decades. Most of the photosensitizers used in cancer therapy are based on a tetrapyrrole structure, similar to that of the protoporphyrin in hemoglobin. Combinations of various therapeutic modalities with non-overlapping toxicities are among the commonly used strategies to improve the therapeutic index of treatments in modern oncology. Sensitization of tumor cells to PDT and interference with cytoprotective molecular responses triggered by PDT in surviving tumor cells increase the antitumor effectiveness. More than 25 years ago, PDT was clinically approved for the treatment of selected tumors 60. A highly selective method for the destruction of unwanted cells and tissues is the main advantage of PDT. PDT not only kills the targeted cells and damage the tumor-associated vasculature but also activate an antitumor immune response 61. The first mechanism of PDT was identified based on the significant variation observed in the level of antioxidant molecules expressed in cancer cells 62. In PTD-based targeting therapies, photosensitizers are covalently attached to various molecules that have some affinity for neoplasia or to receptors expressed on specific tumors 63. The clinical efficacy of PDT is dependent on complex dosimetry of total light dose, light exposure time, and light delivery mode 64. The choice of the light source should, therefore, be based on photosensitizer absorption (fluorescence excitation and action spectra), disease (location, size of lesions, accessibility, and tissue characteristics), cost, and size 65. Lasers can be coupled into fibers with diffusing tips to treat tumors, such as reported in the urinary bladder and digestive tract cases 66, 67. Neo-adjuvant therapy is often given in an attempt to shrink tumors and improve the chance of successful surgery. A study had shown that postoperative PDT could enhance mean survival time compared to standard postoperative care alone 68. In a different study, mesothelioma patients undergoing pleurectomy followed by postoperative PDT showed unusually long survival, most likely due to the preservation of the lung and/or the PDT effect 69, 70. These studies indicate PDT can be easily implemented in standard care regimens, either pre or post-operation, to improve therapy outcomes. Yanovsky and colleagues had reviewed the recent updates in the use of PDT for the treatment of various cancers 8. Advances in basic and clinical research on PTD have given opportunities to improve its efficacy in lung and other cancer treatments 12, 71. Li and colleagues had developed a singlet oxygen responsive micelles-based PDT nano-platform for interactively triggered photosensitizer delivery that improves antitumor PDT efficacy 35, 36. This nano platform showed a robust antitumor effect against lung and breast cancer both in vitro and in vivo. It has been reported that the highly reactive singlet oxygen in PDT could deplete glutathione (GSH) and activate ferroptosis 37. Meng and colleagues fabricated a disulfide-bearing imidazole ligand coordinated with zinc to form an all-active metal-organic framework nanocarrier with the potential to deplete intracellular GSH and enhance PDT antitumor potential 37. However, these PDT nanoplatforms are still at an early stage of translation and need more extensive clinical trials for successful clinical application in the future.

## PDT in lung cancer

PDT for non-small cell lung cancer (NSCLC) was first used in 1982 to achieve tumor necrosis and airway reopening. PDT is considered to be more specific and lesion-oriented compared with other available treatment modalities and produces less collateral damage with fewer complications. PDT was also tried in patients with early central lung cancer when patients were unable to undergo surgery to improve their general life quality. PDT was deemed well-tolerated and effective as part of a multi-modal treatment for non-small cell lung cancer (NSCLC) in a small retrospective study 72. PDT following chemical or radiotherapy can achieve local tumor control for longer periods compared to other modalities or either treatment alone 72. Other studies have also supported these effective results of PDT in lung cancer 3, 16, 73. The palliative efficacy and safety of PDT as part of a multi-modal treatment were evaluated in a single-center prospective pilot study with patients suffering from advanced NSCLC with central airway obstruction by intravenous administration 74. All patients showed improvement in their symptoms with significantly improved lung capacity and function. One year post-PDT, survival was markedly improved for PDT treated patients compared to the one-year survival rate mentioned for patients with NSCLC treated with systemic CT alone 74, 75. A Phase study showed hexyloxyethyl devinylpyropheophorbide-PDT is capable of achieving high rates of chemotherapeutic efficacy retained for months in patients with carcinoma in situ (CIS) and micro-invasive cancer (MIC) of the central airways 76. Two ongoing clinical trials are testing the safety and efficacy of new photosensitizers in lung cancer. One trial is investigating the safety and efficacy of the water-soluble palladium-bacteriochlorophyll WST11 in obstructive NSCLC (EudraCT ID: 2009-011895-31). WST11 had improved the effectiveness compared to older photosensitizers and fewer side effects due to rapid clearance 77. Another study is an open-label Phase II study to evaluate the safety, tolerability, and efficacy of Fotolon ® (Chlorin e6-PVP) for the treatment of obstructing NSCLC (EudraCT ID: 2013-001876-39). In contrast, newer photosensitizers like talaporfin that has higher absorption bands at longer wavelengths show increased efficacy, making them suitable for cases where first-generation Photofrin ® fails (NSCLC, Clinicaltrials.gov ID: NCT02916745). A systemic review by Maziak and colleagues had well documented the use of PDT on NSCLC and its' challenges 78. They concluded that PDT may be most effective for small and superficial airway lesions of <1 cm length. In patients with early-stage lung cancer, PDT has been successfully used to treat patients for whom surgery is not suitable. In one phase II study, 54 patients with lung carcinoma underwent porfimer sodium-mediated PDT and showed an 85% complete response rate with a 6.5% local failure rate at 20.2 months 79. Another typical study had shown a complete response rate of 94% with 80% local control at 5 years 80. A randomized trial of PDT versus Nd: YAG laser therapy for obstructing NSCLC lesions showed equal initial efficacy for these 2 treatments, with a longer duration of response noted for PDT. The guidelines of the American College of Chest Physicians recommended that PDT is only suitable for lesions under 1 cm in diameter based on results with Photofrin ® in 2003. However, no significant difference in efficacy was observed between tumors under or over 1 cm when using talaporfin 81. A recent review by Ikeda N and colleagues concluded the PDT as an effective therapy for central-type early-stage lung cancer (CELC) of < 1 cm diameter 82. Similarly, El-Hussein and colleagues summarized the efficacy of combined use of PDT and chemotherapy in lung cancer treatment 83. Another review by Ohtani and Ikeda suggested that PDT can also be effective for advanced lung cancer, causing tracheobronchial obstruction 84. PDT therapy achieved complete remission in 86.4% of the total number of lesions in 141 patients (191 lesions) with CELC 85.

## Combination and converse effect of PDT in lung cancer

PDT frequently provokes a strong acute inflammatory reaction that can lead to the development of systemic immune response observed as localized edema at the targeted site. The relative contribution of PDT depends to a large extent on the type, and a dose of photosensitizer used, the time between photosensitizer administration and light exposure, total light dose and its fluence rate, tumor oxygen concentration, and perhaps other still poorly recognized variables. The acute inflammatory response is a major protective effect. The inflammation elicited by PDT is a tumor antigen nonspecific process orchestrated by the innate immune system. The onset of PDT-induced inflammation is marked by dramatic changes in the tumor vasculature 61. Photosensitivity is another common complication, which can last for months. In the majority of cases, it is mild-to-moderate and requires no treatment. Visual discomfort is also listed among the side effects of PDT 86. It is important to stress that most side effects can be alleviated by the proper selection of types and dosage of photosensitizer, parameters of illumination, and other details of the PDT treatment protocol. Standardization of the treatment protocols and prediction of the PDT response, however, is seriously hampered by the lack of established PDT dosimetry 42. In contrast to ionizing radiation, no agreement has been reached on how the doses of PS and light should be measured, and even no widely accepted definition of dose exists. Besides, the optimum PS and light doses, as well as drug-light time interval, may vary from patient to patient or lesion to lesion, which prevents the application of standardized protocols and the achievement of the highest response rates. Among the limitations of PSs currently used for clinical PDT are the difficulty in treating large tumor masses and the limited depth of treatment. Visible light can penetrate the tissues not deeper than 5-10 mm, which restricts the application of PDT to mainly superficial lesions. A detailed description of the current state of PDT and its limitations can be found in comprehensive reviews 42, 86.

## Advantages and disadvantages of PDT in lung cancer

Over conventional lung cancer treatment approaches, PDT has several advantages such as less invasive procedure compared to surgical resection, more target-specific, minimum adverse effect on surrounding healthy tissues, negligible systemic adverse effects, and cost-effectiveness. PDT is convenient to administer, can be applied in outpatient setup, and possible to apply multiple times on the desired location without leaving scars after healing. Both the photosensitizer and visible light source are not as toxic as the chemotherapies or radiotherapies. PDT can be used in combination with other cancer therapies, including radiotherapy, chemotherapy, and photothermal therapy.

Similar to other therapies for lung cancer, PDT also holds limitations. Low tissue penetration properties of visible light used in PDT causes difficulties to treat deep-seated tumors in lung tissue. Similarly, a limited amount of oxygen in tumor tissues surrounded by dense necrotic tissue and tumor masses can reduce the effectiveness of PDT. Commonly available photosensitizers for lung cancer treatment are non-specific to cancer cells. PDT is mainly for localized cancer and generally cannot be used to treat metastasized cancer 87. Although the adverse effect of PDT on the surrounding tissue is minimal, PDT can cause a burn, swelling, and scarring in the nearby normal tissues 88. PDT of lung cancer causes temporary side effects such as coughing, painful breathing, or shortness of breath. The waiting time of photosensitizer administration to laser light illumination is called a drug-light interval. The drug-light interval 24-96 h is another limitation of PDT. However, the use of vascular-targeted photosensitizers such as chlorine e6 has reduced the drug-light interval time to 3 h with promising results in NSCLC treatment 89.

## The trend and future direction

Development of efficient photosensitizers that can more specifically target cancer cells, and approaches to deliver light that can penetrate large or deep tumor tissue should be developed. Photosensitizers conjugated with specific nanoparticle platform could be developed for better penetration. Moreover, nanoplatforms functionalized with specific receptor-based detectors such as antibody constructs, monoclonal antibodies, or small molecules inhibitors could improve lung cancer cell-specific delivery of photosensitizers 34. The design of innovative equipment to improve the delivery of light sources is urgently needed for the higher efficacy of PDT in large or deep lung cancer. Pieces of literature have suggested that PDT triggers the immune response to control local and metastasized cancer. The beneficial role of intraoperative PDT on mesothelioma patients might be via modulation in immune response 90. Therefore, the development of a novel combination of PDT and immune checkpoint blockade therapy could be beneficial for metastatic lung cancer 91. Recently, Yang and colleagues developed a sequential PDT and photothermal using Gd-Ce6@SWNHs platform with cooperative and long-lasting antitumor immune responses for the treatment of patients with advanced metastatic cancer 92. The application of novel PDT platforms, nanoparticle-based photosensitizers, and improved imaging and surveillance system is crucial to improve the efficacy of PDT in lung cancer treatment.

## Conclusions

As a stand-alone treatment, PDT proved an alternative for palliative chemotherapy or radiotherapy in unresectable lung cancer as it achieved an overall response of nearly 87% and improved patient quality of life. PDT is still considered to be a new and promising antitumor strategy. The advantages of PDT compared with surgery, chemotherapy, or radiotherapy are reduced long-term morbidity and the fact that PDT does not compromise future treatment options for patients with recurrent disease. Thus, the application of PDT for lung cancer treatment needs to be further evaluated in extensive clinical trials.
